# Revolutionary drug repositioning: the preventive and therapeutic potential of metformin and other antidiabetic drugs in age-related macular degeneration

**DOI:** 10.3389/fphar.2024.1507860

**Published:** 2024-12-10

**Authors:** Yating Zhou, Fei Xue

**Affiliations:** Kunshan Hospital of Traditional Chinese Medicine, Suzhou, Jiangsu, China

**Keywords:** age-related macular degeneration (AMD), antidiabetic drugs, metformin, AMPK activation, precision medicine

## Abstract

Age-related macular degeneration (AMD) is a leading cause of blindness among the elderly worldwide. Anti-vascular endothelial growth factor (anti-VEGF) injections remain the first-line therapy for AMD. However, their high cost and the need for frequent administration pose challenges to long-term adherence, highlighting the need for accessible and cost-effective preventive strategies. Emerging evidence suggests that traditional antidiabetic drugs, such as metformin, sulfonylureas, and thiazolidinediones, may offer neuroprotective benefits, opening new avenues for AMD prevention. Among these, metformin has emerged as the most promising candidate, demonstrating significant potential in reducing AMD risk, even at low cumulative doses, primarily through AMP-activated protein kinase (AMPK) activation. Sulfonylureas, although effective in stimulating insulin secretion, carry risks such as hypoglycemia, hyperinsulinemia, and a possible association with increased cancer risk. Similarly, thiazolidinediones, while improving insulin sensitivity, are associated with adverse effects, including cardiovascular risks and macular edema, limiting their broader application in AMD prevention. This paper explores the preventive potential and underlying mechanisms of these antidiabetic drugs in AMD and discusses the role of artificial intelligence in optimizing individualized prevention strategies. By advancing precision medicine, these approaches may improve public health outcomes and reduce the burden of aging-related vision loss.

## 1 Introduction

### 1.1 The global challenge of AMD: an unresolved issue

Age-related macular degeneration (AMD) is a degenerative eye disease primarily affecting individuals aged 55 years and older, and it is a leading cause of irreversible vision loss in developed countries (Li et al., 2020). Globally, approximately 8.7% of the population is affected by AMD, with an estimated 196 million patients in 2020, projected to increase to 288 million by 2040 (Wong et al., 2014). Late-stage AMD includes neovascular (wet) and geographic atrophy (late dry, GA) forms, both of which are closely associated with significant vision loss. Major risk factors include smoking, poor nutrition, cardiovascular disease, and genetic predisposition (Lim et al., 2012; de Jong et al., 2020). Early symptoms of AMD include blurry vision, central vision loss, and distorted lines, which may ultimately lead to complete central vision loss. These impairments severely impact daily life and increase the risk of mental health issues such as anxiety, depression, and social isolation (Gheorghe et al., 2015; Hwang et al., 2023a).

From an economic perspective, the treatment costs of AMD impose a significant burden on individuals, families, and society. Neovascular AMD is a primary cause of irreversible vision loss, with patients incurring an average cost of €17,265 in the first year post-diagnosis, primarily attributed to direct medical expenses (Abraldes et al., 2024). Although anti-vascular endothelial growth factor (anti-VEGF) therapy is the current mainstay of treatment, its high cost and the need for frequent injections make it difficult for many patients, particularly those with lower incomes, to maintain long-term treatment adherence (Brown et al., 2021; Spooner et al., 2018). Therefore, there is an urgent need to develop new, cost-effective, and broadly applicable treatment and prevention strategies, especially given the growing patient population and increasing aging demographic.

### 1.2 The unexpected potential of antidiabetic drugs: a possible game changer

In recent years, traditional antidiabetic drugs have shown potential in treating a variety of diseases, prompting renewed attention from the academic community. Diabetes, especially type 2 diabetes, is considered a potential risk factor for AMD (Hwang et al., 2023b; Chen et al., 2014). Diabetes-induced oxidative stress, chronic inflammation, and the accumulation of advanced glycation end products may contribute to the development of AMD by damaging the retinal pigment epithelium (RPE) and endothelial cells (Jadeja and Martin, 2021; Tian et al., 2005; Dionysopoulou et al., 2023; Amato et al., 2021).

In this context, antidiabetic drugs, particularly metformin, have garnered increasing attention for their potential in preventing AMD progression. Research indicates that metformin may offer neuroprotection by improving metabolic status and reducing inflammation (Romdhoniyyah et al., 2021; Francisco and Rowan, 2023; Du et al., 2022; Brown et al., 2019), especially in high-risk elderly populations, thereby delaying disease progression and enhancing quality of life (Holtz et al., 2023; Amin et al., 2022; Khanna et al., 2022; Kaufmann et al., 2023). Other antidiabetic drugs, such as sulfonylureas and thiazolidinediones, have also shown potential for AMD prevention in early studies (Francisco and Rowan, 2023; Picard et al., 2024). These findings suggest that drug repurposing may provide new preventive pathways and offer a more cost-effective solution for AMD patients. Additionally, novel delivery systems, such as lipid-based nanoparticles, may enhance ocular bioavailability and support the application of antidiabetic drugs in targeting the posterior segment of the eye (Puglia et al., 2021). Therefore, this paper will further explore the potential and practical applications of these antidiabetic drugs in AMD prevention and treatment.

## 2 Multifunctional mechanisms of diabetes drugs: from glucose lowering to retinal protection

### 2.1 Retinal protective effects of metformin: an affordable star drug

Metformin, a traditional antidiabetic drug, has garnered increasing attention in recent years for its potential protective effects against AMD. These protective effects involve several interrelated mechanisms. Firstly, metformin activates the AMP-activated protein kinase (AMPK) pathway, which plays a key role in protecting RPE cells by inhibiting oxidative stress and inflammation (Datta et al., 2017; Xu et al., 2018). AMPK activation inhibits Mechanistic target of rapamycin (mTOR) and activates the Unc-51-like kinase (ULK) complex, initiating autophagy to clear damaged organelles and decrease reactive oxygen species (ROS) This leads to the inhibition of NOD, LRR and pyrin domain-containing protein 3 (NLRP3) inflammasome activation, thereby protecting RPE cells (Meyer et al., 2019; Zhao et al., 2020; Yang et al., 2019). Furthermore, metformin activates mitophagy, reducing mitochondrial ROS (mtROS) and chronic inflammation (Lu et al., 2021; Toppila et al., 2024). Additionally, AMPK activation also enhances mitochondrial function by improving oxidative phosphorylation, restoring ATP levels, and meeting the high metabolic demands of RPE cells, thus maintaining mitochondrial homeostasis (Xu et al., 2018; Dieguez et al., 2024). Through these combined mechanisms, metformin protects RPE cells from oxidative damage and is hypothesized to slow the progression of AMD.

**FIGURE 1:**
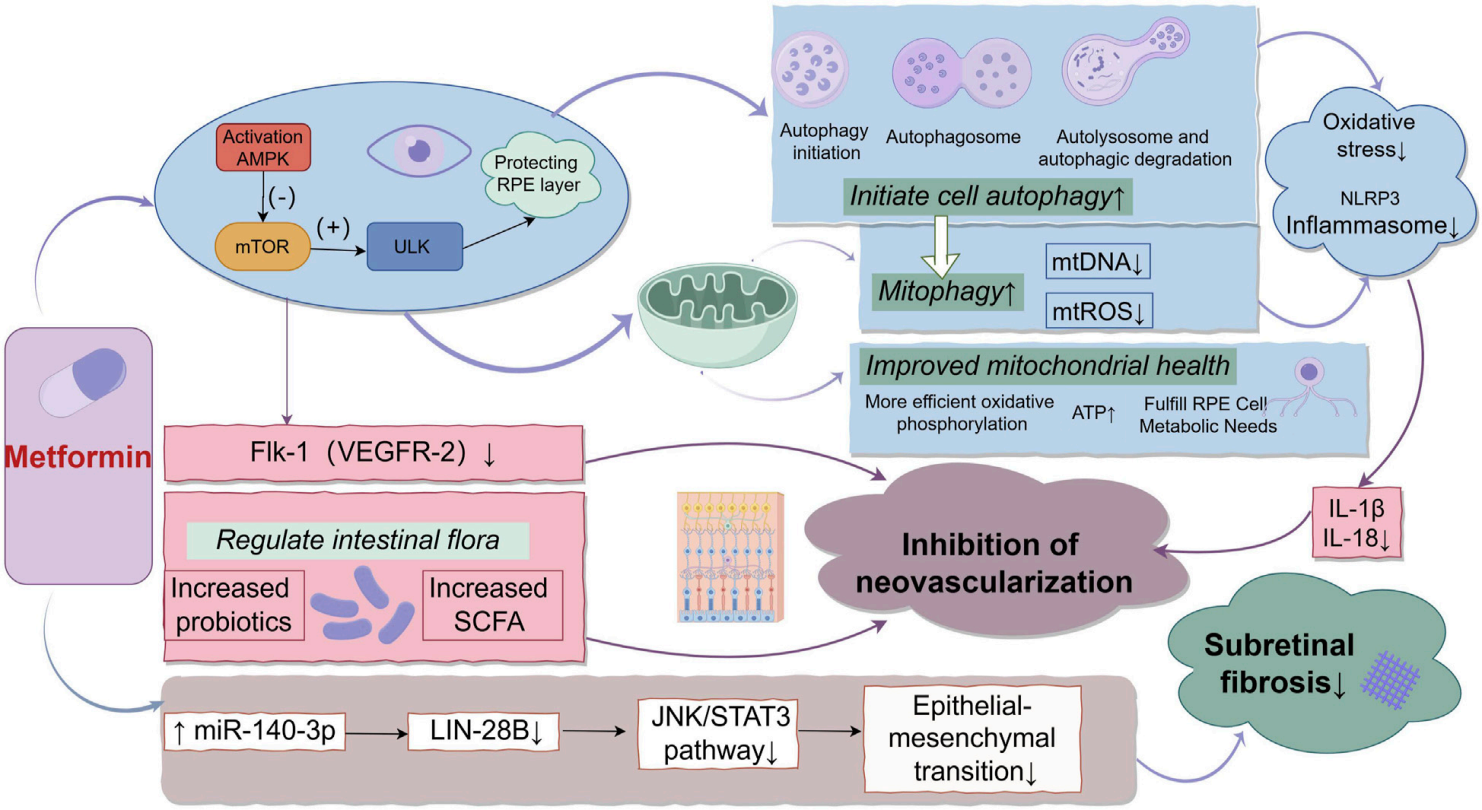
The Multifaceted Mechanisms of Metformin in AMD. Illustrates the multifaceted mechanisms of metformin in the treatment of AMD. Metformin activates the AMPK pathway, which inhibits oxidative stress and inflammation. The activation of AMPK initiates both cellular and mitochondrial autophagy, reducing the release of mitochondrial DNA and ROS, thereby inhibiting NLRP3 inflammasome activation and protecting RPE cells. In addition, AMPK improves mitochondrial function, restores ATP levels, and meets the high metabolic demands of RPE cells. Metformin also inhibits pathological neovascularization by downregulating the VEGF receptor (Flk-1/VEGFR-2). Furthermore, it modulates the gut microbiome, increasing SCFA production, which further suppresses retinal neovascularization. Lastly, metformin upregulates miR-140-3p, suppresses LIN28B activity, and inhibits the JNK/STAT3 pathway, thereby reducing EMT and inhibiting subretinal fibrosis.

One of the pathological features of advanced AMD is vascular endothelial growth factor (VEGF)-driven neovascularization (Ohno-Matsui et al., 2001). Studies have shown that metformin inhibits pathological neovascularization by downregulating the VEGF receptor Flk1, which has potential benefits for preventing or treating nAMD (Joe et al., 2015; Han et al., 2018). However, the effect of metformin on angiogenesis is inconsistent across studies, potentially due to tissue-specific differences, necessitating further research to clarify these mechanisms (Dallaglio et al., 2014). Recent studies have also shown that metformin indirectly inhibits neovascularization by modulating the gut microbiome (Zhang et al., 2023). Metformin increases the abundance of Bifidobacterium and Akkermansia and promotes the production of short-chain fatty acids, thereby reducing pathological retinal neovascularization through the “gut-retina axis.” In addition to neovascularization, epithelial-mesenchymal transition (EMT) is another critical pathological process in late-stage AMD that is associated with subretinal fibrosis (Wu et al., 2022; Shu et al., 2020). Metformin inhibits EMT by upregulating microRNA-140-3p, suppressing Lin-28 Homolog B activity, and consequently downregulating the JNK/STAT3 pathway, reducing fibrosis (Hua et al., 2023; Mitra et al., 2024; Wang et al., 2021). The detailed mechanisms are illustrated in Figure 1.

In addition to the support from mechanistic studies, recent epidemiological research has also provided evidence for the preventive role of metformin in reducing the risk of AMD. Multiple studies, as summarized in Table 1, have demonstrated a significant association between metformin use and reduced AMD risk, with some studies highlighting a dose-response relationship (Khanna et al., 2022; Kaufmann et al., 2023; Moir et al., 2024; Aggarwal et al., 2024; Jiang et al., 2022; Tseng, 2023). Furthermore, a recent meta-analysis integrating multiple studies has further supported this protective trend (Holtz et al., 2023). However, some studies have not found a significant effect of metformin on AMD risk, indicating that, while the overall evidence leans positive, the heterogeneity among study results warrants attention (Shen et al., 2024; Eton et al., 2022; Huang et al., 2023). Recent epidemiological research has predominantly supported the preventive role of metformin in AMD, while its potential therapeutic effects remain under investigation. Therefore, larger-scale, well-designed prospective studies are needed to clarify the actual efficacy of metformin in AMD prevention and treatment.

**TABLE 1:** Metformin use and risk of AMD: Summary of evidence.

Author(s)/Country/Region	Study type	Database	Age	Diagnostic criteria	Sample size	AMD risk (OR/HR)	Conclusion
Moir et al. (2024), United States	Observational Cohort	Merative MarketScan Database	≥60	ICD-11	Total: 21,007 (Non-diabetic: 15,219)	OR = 0.88 (95% CI: 0.79–0.99)	Positive—Metformin reduces GA risk
Khanna et al. (2022), United States	Case-Control Study	Merative MarketScan Database	≥55	ICD-9, ICD-10	Total: 173,848	OR = 0.95 (95% CI: 0.91–0.98)	Positive—Metformin reduces AMD risk
Aggarwal et al. (2024), United States	Case-Control Study	Merative MarketScan Database	≥55	ICD-10	Total: 464,021 participants (Non-diabetic)	OR = 0.83 (95% CI: 0.74–0.87)	Positive—Reduced AMD risk in non-diabetic
Kaufmann et al. (2023), United States	Case-Control Study	Merative MarketScan Database	≥55	ICD-9, ICD-10	Total: 388,125 (Diabetic: 99,448)	OR = 0.97 (95% CI: 0.95–0.99)	Positive—Reduced dry AMD risk
Jiang et al. (2022), China	Retrospective Study	Hospital Records	≥50	ICD-10	Total: 324	OR = 0.23 (95% CI: 0.13–0.38)	Positive—Significant reduction in AMD risk
Tseng (2023), Taiwan	Retrospective Cohort	Taiwan National Health Insurance	50–79	ICD-9-CM	Total: 26,606 (Ever Users: 13,303, Never Users: 13,303)	HR = 0.756 (95% CI: 0.673–0.850)	Positive—Significant reduction in AMD risk
Shen et al. (2024), United States	Randomized Phase II	Multi-center Study	≥55	Image-based Diagnosis of GA	Total: 66 participants (Non-diabetic)	Rate Difference = 0.07 mm/year (95% CI: −0.05 to 0.18, *p* = 0.26)	Neutral—No significant effect on GA progression
Elhalag et al. (2024), United States	Meta-analysis	PubMed, Scopus, Web of Science	Unlimited	Various	Total: 1,447,470 patients (Diabetic)	OR = 0.37 (95% CI: 0.14–1.02), *p* = 0.05	Neutral—No significant difference
Huang et al. (2023a), Taiwan	Cohort Study	Taiwan National Health Insurance	≥50	ICD-9, ICD-10	Total: 728,703 new (Diabetic)	OR = 0.93 (<5 defined daily dose/month); OR = 1.39 (>25 defined daily dose/month)	Neutral—Dose-dependent association with AMD risk
Eton et al. (2022), United States	Retrospective Cohort	Clinformatics^TM^ Database	≥55	ICD-9, ICD-10	Total: 1,007,226 (Diabetic)	Current Users: HR = 1.08 (95% CI: 1.04–1.12); Prior Users: HR = 0.95 (95% CI: 0.92–0.98)	Neutral—Conflicting associations observed

### 2.2 Neuroprotective effects of sulfonylureas: untapped potential

Glibenclamide, a traditional sulfonylurea used to control blood glucose levels in type 2 diabetes, has recently attracted attention for its neuroprotective effects in the retina. By targeting sulfonylurea receptor 1 (SUR1) and co-localizing with potassium channels (Kir6.2) and cation channels (TRPM4), glibenclamide modulates ion flow across the cell membrane, reducing cell depolarization and effectively mitigating oxidative stress-induced damage to RPE cells. Additionally, it inhibits the activation of the NLRP3 inflammasome, thereby reducing chronic inflammatory responses (Berdugo et al., 2021; Zhang et al., 2017; He et al., 2022), improving retinal cell function, balancing growth factors, and reducing retinal damage (Wong et al., 2014) as well as extending the lifespan of retinal ganglion cells (Conti et al., 2022; Chou et al., 2018). Recent *in vitro* experiments and case-control studies have demonstrated that glibenclamide protects cone cells from oxidative stress and apoptosis, thereby lowering the risk of developing late-stage dry AMD (Picard et al., 2024). However, large-scale clinical trials are currently lacking, and further research is needed to determine the practical application and feasibility of glibenclamide in AMD prevention and treatment.

### 2.3 Thiazolidinediones: a double-edged sword for retinal protection

Thiazolidinediones (TZDs), such as rosiglitazone and pioglitazone, are Peroxisome proliferator-activated receptor gamma (PPARγ) agonists initially used to control blood glucose levels in type 2 diabetes but have shown complex effects in AMD. TZDs can inhibit VEGF gene promoter activity, reducing VEGF expression and suppressing neovascularization (Peeters et al., 2006). However, they may also increase VEGF levels, leading to vascular leakage and new vessel formation (Ku et al., 2017). Additionally, TZDs reduce chronic inflammation by inhibiting Tumor necrosis factor-alpha (TNF-α) (Carta et al., 2011). Due to tissue-specific effects, the impact of TZDs can vary across different pathological conditions. A 2-year study found that patients using TZDs experienced a reduction in subretinal fluid after anti-VEGF treatment, but with an associated increased risk of intraretinal fluid (IRF), indicating the need for further investigation into their long-term effects (Core et al., 2023).

## 3 The prospects of drug repurposing: overcoming the barriers of indications

### 3.1 Drug repurposing: bridging endocrinology and ophthalmology

Drug repurposing, the application of approved drugs to new indications, has gained significant attention in recent years. Its advantages include shortening drug development timelines, reducing costs and risks, and accelerating clinical translation to benefit more patients (Pushpakom et al., 2019). Classic examples include Viagra (originally developed for cardiovascular conditions but later used for erectile dysfunction and pulmonary hypertension) (Ghofrani et al., 2006), and Thalidomide (repurposed from a morning sickness treatment to a therapy for leprosy and multiple myeloma) (Wang et al., 2016). The repurposing of diabetes drugs, such as metformin, holds the potential to offer a more affordable and accessible treatment option for patients. This interdisciplinary approach shows promise in providing safe and effective strategies for managing retinal degenerative diseases, including AMD.

### 3.2 Exploring applications beyond diabetes: a revolutionary approach

A bold but worth-exploring question is whether metformin could be integrated into health management plans for high-risk elderly populations to prevent AMD. Similar to the widespread use of aspirin in cardiovascular prevention, metformin’s potential preventive effects in non-diabetic populations are gaining attention. A recent study in JAMA Ophthalmology found that metformin use was associated with a reduced risk of AMD, even in patients without diabetes (Aggarwal et al., 2024). However, it is important to interpret this finding with caution. Most evidence supporting metformin’s role in AMD prevention, including this study, comes from observational data, which may not entirely exclude the possibility of undiagnosed diabetes among registered metformin users. Since metformin is primarily prescribed for type 2 diabetes, it is likely that some of these users were in prediabetic or early diabetic stages. This limitation highlights the necessity of future prospective studies to validate metformin’s independent preventive effects in strictly non-diabetic populations and to elucidate its underlying mechanisms.

In comparison, the potential of sulfonylureas and TZDs for AMD prevention is more limited. Sulfonylureas, though effective in stimulating insulin secretion, are associated with higher risks of hypoglycemia, hyperinsulinemia (Tseng and Tai, 1992), and possibly cancer (Hsieh et al., 2012). TZDs, while improving insulin resistance, carry risks such as cardiovascular events with rosiglitazone and bladder cancer with pioglitazone (Tseng, 2012). Their association with macular edema further restricts their potential use in retinal disease prevention (Nien and Tseng, 2014).

Metformin’s anti-inflammatory and antioxidant properties not only support AMD prevention but also suggest broader benefits, including anti-aging, cardiovascular protection, cancer prevention, and depression management (Kulkarni et al., 2020; Dihoum et al., 2023; Yao et al., 2024; Ríos et al., 2024; Syed et al., 2022). Additionally, epidemiological studies indicate metformin may reduce risks of dementia (Chin-Hsiao, 2019), hypertension (Tseng, 2018), atrial fibrillation (Tseng, 2021a), heart failure (Tseng, 2019), and inflammatory bowel disease (Tseng, 2021b). These findings highlight metformin’s unique value across multiple fields, supporting its potential as a widely applicable preventive medication.

## 4 Safety, controversies, and risks

### 4.1 Long-term use in non-diabetic populations: side effects and solutions

While metformin shows potential in preventing AMD in non-diabetic populations, its long-term use raises some safety concerns. The most common side effects are gastrointestinal issues, including diarrhea, nausea, and abdominal discomfort, particularly during the early stages of treatment (Bonnet and Scheen, 2017). Another key concern is vitamin B12 deficiency, which may lead to anemia and neurological symptoms, especially in elderly patients (Infante et al., 2021). Regular monitoring of vitamin B12 levels and supplementation when necessary is recommended for patients on long-term metformin therapy (Shahjahan et al., 2024). Although rare, there is a risk of lactic acidosis, particularly in patients with impaired liver or kidney function (Visconti et al., 2016). Notably, recent studies have highlighted that type 2 diabetes patients hospitalized for heart failure and/or acute coronary syndrome may face an elevated risk of metformin-related lactic acidosis, which, though infrequent, can be fatal (Tseng, 2024). This underscores the importance of careful patient selection and monitoring when prescribing metformin in populations with comorbidities.

Genetic testing can identify individuals most likely to benefit from metformin while minimizing side effect risks. Variations in organic cation transporter 1 (OCT1), encoded by the SLC22A1 gene, significantly influence metformin absorption and efficacy (Chan et al., 2018). For instance, the rs72552763 (Met420del) variant reduces drug uptake, increasing gastrointestinal side effects, while rs628031 (Met408Val) may lower OCT1 expression, affecting absorption and efficacy differently across populations (Aladhab et al., 2023; Mato et al., 2018). Incorporating genetic testing into clinical practice enables targeted therapy by identifying high-risk individuals, optimizing metformin use, and supporting personalized strategies, particularly for AMD prevention.

### 4.2 Should metformin be combined with anti-VEGF therapy?

Current research on metformin primarily focuses on its preventive effects against AMD; however, a few studies have begun to explore its potential in treating AMD (Ebeling et al., 2022; Luo et al., 2021). Anti-VEGF therapy is the current standard treatment for wet AMD, significantly improving vision by inhibiting the growth of pathological neovascularization (Amoaku et al., 2015). While there is no direct evidence supporting metformin as a standalone treatment for AMD, findings from diabetic macular edema (DME) research provide valuable insights. For instance, one study reported that combining metformin with anti-VEGF therapy significantly improved visual acuity and central macular thickness in DME patients, while reducing the frequency of anti-VEGF injections (Shao et al., 2022). Another study suggested that metformin may enhance vision recovery, reduce retinal thickness, and mitigate anti-VEGF resistance (Uwimana et al., 2022). Although these findings hint at a potential auxiliary role for metformin in AMD treatment, they are primarily derived from DME studies. High-quality clinical trials are needed to determine whether similar benefits can be observed in AMD patients. Future research should also clarify the underlying mechanisms and assess the safety and efficacy of metformin when used in combination with anti-VEGF therapy.

## 5 Future directions: establishing a new framework for AMD prevention

### 5.1 Personalized treatment and AI optimization

Future research should focus on identifying which patients are most likely to benefit from metformin for AMD prevention. Additionally, its potential role in treatment for certain AMD subtypes could also be explored. By integrating patient genetic profiles, inflammation levels, and other biomarkers with the chemical properties of the drug, AI (Artificial Intelligence) can combine genetic or proteomic data with chemical structures to score treatment effectiveness, helping to select patients who are most likely to respond favorably (Romm and Tsigelny, 2020). Additionally, AI can predict drug interactions based on structural and target similarities, optimizing dosage regimens to maximize efficacy (Chen et al., 2023). These AI-driven approaches will contribute to building personalized treatment models, shifting AMD management from “one-size-fits-all” to precision medicine.

### 5.2 The necessity of clinical trials and multidisciplinary collaboration

The successful implementation of drug repurposing requires close interdisciplinary collaboration. Experts in ophthalmology, endocrinology, and public health should work together to design clinical trials that assess the efficacy of metformin for AMD prevention in diverse populations. Multicenter collaborations can ensure that the treatment is applicable to a broad patient base, particularly in resource-limited regions. Additionally, the involvement of social scientists can effectively evaluate the societal acceptance and cost-effectiveness of repurposed drugs, facilitating global adoption.

### 5.3 Integrating metformin into elderly health management

Incorporating metformin into elderly health management as a preventive medication could be a promising future strategy. For elderly individuals with high AMD risk factors (e.g., family history, smoking, malnutrition), metformin may play a critical role in reducing AMD risk and slowing disease progression. Early intervention with metformin could not only lower AMD incidence but also reduce healthcare costs. Governments and health organizations should support related research and develop guidelines to implement this preventive strategy, achieving true “prevention before disease” in public health.

## 6 Conclusion

The application of metformin has extended beyond diabetes management, with recent studies highlighting its unique advantages in AMD prevention and treatment. This paper explores the strategy of drug repurposing, positioning this “cost-effective” medication as a solution to the challenge of vision loss in AMD. Compared to existing high-cost treatments, the cross-application of metformin, especially in resource-limited areas, may offer a more affordable alternative. While research on sulfonylureas and TZDs in AMD prevention remains preliminary, their neuroprotective effects provide important directions for future study. Additionally, integrating AI to predict drug selection and individual responses could help advance precision medicine in ophthalmology. Overall, metformin not only offers new hope for AMD patients but also presents a novel opportunity for health management in an aging society.

## Data Availability

The original contributions presented in the study are included in the article/supplementary material, further inquiries can be directed to the corresponding author.
